# A circulating cell-free DNA methylation signature for the detection of hepatocellular carcinoma

**DOI:** 10.1186/s12943-023-01872-1

**Published:** 2023-10-06

**Authors:** Si-Cho Kim, Da-Won Kim, Eun Ju Cho, Jin-Young Lee, Jiwon Kim, Chaesun Kwon, Jeongsil Kim-Ha, Suk Kyun Hong, YoungRok Choi, Nam-Joon Yi, Kwang-Woong Lee, Kyung-Suk Suh, Won Kim, Woojin Kim, Hyunsoo Kim, Yoon Jun Kim, Jung-Hwan Yoon, Su Jong Yu, Young-Joon Kim

**Affiliations:** 1 Interdisciplinary Program of Integrated OMICS for Biomedical Science, Yonsei University, Seoul, Republic of Korea; 2 R&D center, LepiDyne Inc, Seoul, Republic of Korea; 3 Department of Internal Medicine and Liver Research Institute, Seoul National University College of Medicine, Seoul, 03080 Republic of Korea; 4 Department of Biochemistry, College of Life Science and Biotechnology, Yonsei University, Seoul, 03722 Republic of Korea; 5 Department of Integrative Bioscience & Biotechnology, College of Life Sciences, Sejong University, Seoul, Republic of Korea; 6 Department of Surgery, Seoul National University College of Medicine, Seoul, Republic of Korea; 7 Department of Internal Medicine, Seoul National University College of Medicine, Seoul Metropolitan Government Boramae Medical Center, Seoul, Republic of Korea; 8 Department of Bio-AI convergence, Chungnam National University, Daejeon, Republic of Korea; 9 Department of Convergent Bioscience and Informatics, Chungnam National University, Daejeon, Republic of Korea

**Keywords:** Hepatocellular carcinoma, Methylation-sensitive high-resolution melting analysis, Liquid biopsy, Cell-free DNA, Biomarker, Cancer diagnosis, DNA methylation.

## Abstract

To address the shortcomings of current hepatocellular carcinoma (HCC) surveillance tests, we set out to find HCC-specific methylation markers and develop a highly sensitive polymerase chain reaction (PCR)-based method to detect them in circulating cell-free DNA (cfDNA). The analysis of large methylome data revealed that Ring Finger Protein 135 (RNF135) and Lactate Dehydrogenase B (LDHB) are universally applicable HCC methylation markers with no discernible methylation level detected in any other tissue types. These markers were used to develop Methylation Sensitive High-Resolution Analysis (MS-HRM), and their diagnostic accuracy was tested using cfDNA from healthy, at-risk, and HCC patients. The combined MS-HRM RNF135 and LDHB analysis detected 57% of HCC, outperforming the alpha-fetoprotein (AFP) test’s sensitivity of 45% at comparable specificity. Furthermore, when used with the AFP test, the methylation assay can detect 70% of HCC. Our findings suggest that the cfDNA methylation assay could be used for HCC liquid biopsy.

**Supplementary Information:**

The online version contains supplementary material available at 10.1186/s12943-023-01872-1.

## Main text

To combat the rising incidence rates of hepatocellular carcinoma (HCC), improved detection methods are required [1]. In current monitoring techniques, ultrasound is frequently used in conjunction with serum alpha-fetoprotein (AFP) levels [2]. However, AFP is ineffective in detecting early-stage HCC and frequently produces false positive results in people with active hepatitis [3]. Furthermore, ultrasonography has limitations in detecting early-stage tumors less than 2 cm in size [4]. As a result, there is an urgent need to identify sensitive and specific biomarkers that allow for early detection of HCC.

DNA methylation, in particular, is important in carcinogenesis and has the potential to be used as a cancer diagnostic biomarker [5–7]. The ability to detect liver tumors in blood using DNA methylation markers has shown promise [8–10]. Earlier research, on the other hand, frequently ignored the relevance of DNA methylation patterns in other disease-associated states, resulting in a lack of specificity, particularly in pathogenic liver disorders or other cancer-related cases. Furthermore, because previous research relied heavily on healthy, normal volunteers as controls, the use of cell-free DNA (cfDNA) methylation markers for monitoring in the at-risk cirrhotic population has been limited.

In this study, we describe a machine learning-based cancer biomarker discovery method that uses DNA methylation patterns from various cancer types. We identified Ring Finger Protein 135 (RNF135) and Lactate Dehydrogenase B (LDHB) as distinct methylation markers for HCC and confirmed their clinical efficacy in patients of various racial and etiological backgrounds. To turn these discoveries into a useful diagnostic tool, we developed Methylation-Sensitive High-Resolution Melting (MS-HRM) analysis. Clinical validation of the assay using blood from HCC patients and control people, the combined RNF135 and LDHB MS-HRM analysis was more sensitive than the AFP test in detecting HCC. When used in conjunction with the AFP test, it detected 58% of patients with early-stage HCC (Barcelona Clinic Liver Cancer stage 0-A), demonstrating its efficacy in detecting HCC even at an early stage. Our findings suggest that methylation marker analysis in blood can be used to detect HCC.

## Results and discussion

### Study design

**Fig. 1:**
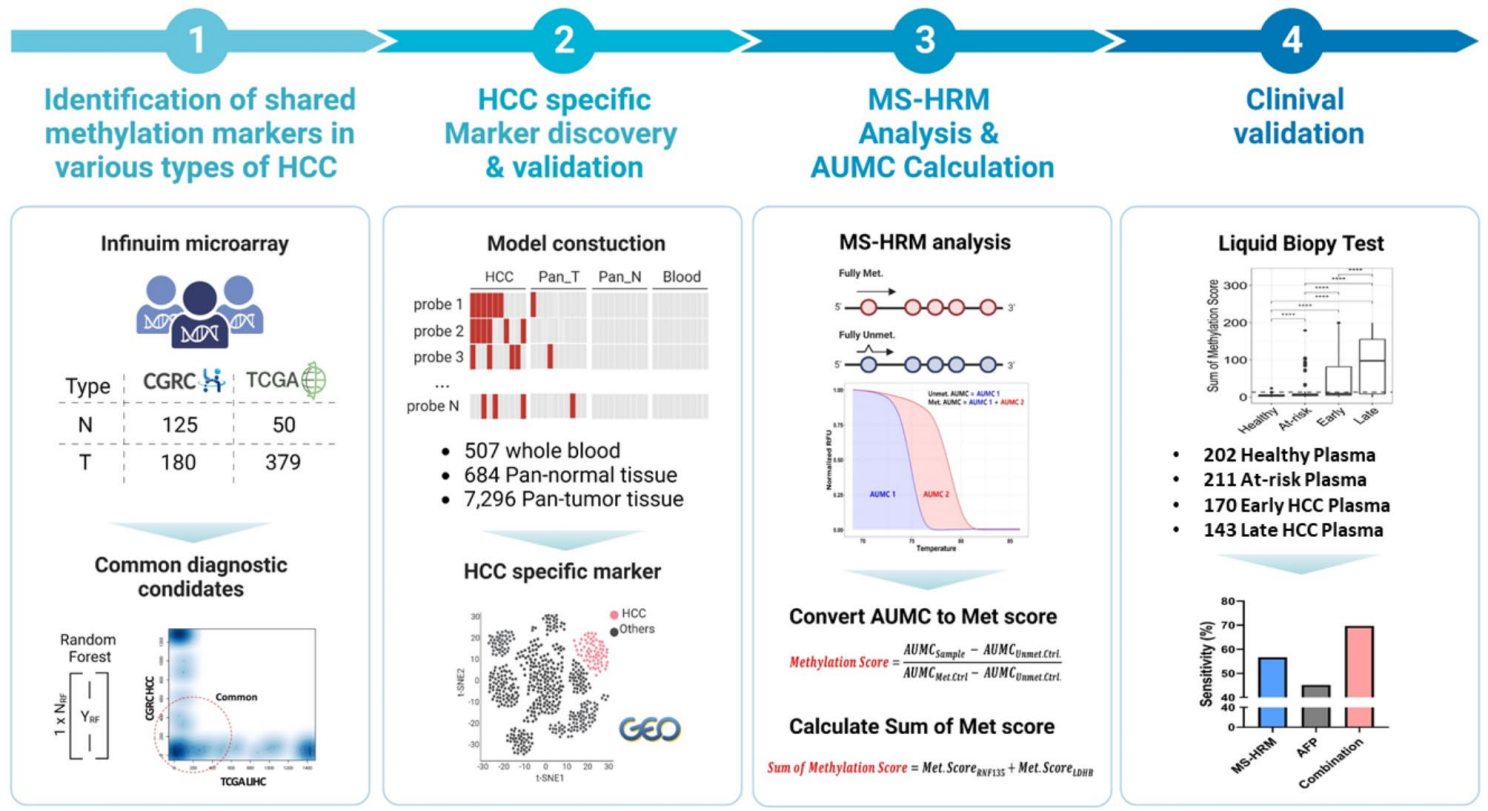
Study Overview. The overall strategy used to develop HCC-specific MS-HRM assay is shown. The first panel displays the number of normal (N) and HCC (T) methylome data from CGRC and TCGA cohorts obtained using Infinium microarray (top). The scatter plot of the Random Forest classification importance rank for markers in two cohorts is shown, with the common markers labeled with a red dotted circle (bottom). The second panel depicts the random forest models used to select HCC-specific markers and the number of samples used to build the model (top), as well as a t-SNE plot that distinguishes HCC from other cell types when clustered with the selected methylation markers (bottom). The third panel shows the CpG sites differentially methylated and their melting curves from MS-HRM analysis (top). The formulas for the methylation score and the sum of methylation score are shown below. The last panel shows the number of blood samples used for the HCC liquid biopsy test and the test results in bar graph along with the sensitivity of the assays shown

Figure 1 and the Supplementary Materials and Methods depict the research design, which was divided into four stages. Initially, we discovered cancer-associated methylation markers that were shared by various types of HCC. Following that, HCC-specific biomarkers were carefully selected based on a meta-analysis of several methylation patterns obtained from other types of cancer and normal tissues. The next step was to develop PCR assays that were specifically designed to detect these methylation markers in blood samples. Finally, blood samples were collected from a diverse group of people, including those who were healthy, those who were at risk, and those who had been diagnosed with HCC at various stages of the disease. These blood samples were then tested for clinical accuracy in detecting HCC using the methods we developed.

### The discovery of universal HCC-specific methylation markers

Our goal was to find biomarkers that could be used in liquid biopsy for HCC. To that end, we examined data from two cohorts: the Cancer Genome Research Center (CGRC) cohort (180 samples; 98.9% Asians), and The Cancer Genome Atlas (TCGA) cohort (379 samples; 49.3% White, 42.2% Asian, and 4.5% Black), which has a more diverse ethnic makeup. Given the high proportion of early-stage HCC in the analyzed cohorts (CGRC: 85%; TCGA: 73.6%), we sought markers that could be used across all stages of cancer, with a focus on markers that would be effective from the disease’s early stages (Table S1). First, we looked for cancer-associated methylation differences (differential methylation (DM) value ≥ 0.3 in ≥ 30% of HCC patients) in each cohort and identified 17,141 and 66,412 differentially methylated probes (DMPs) in the CGRC and TCGA cohorts, respectively. Following that, we removed markers with high methylation levels in blood leukocytes (β-value ≤ 0.15 in ≥ 95% of each cohort), yielding 1,287 and 1,426 probes in each cohort, respectively. The top 100 DMPs distinguishing HCC from nontumor liver and blood samples were then chosen from each cohort using a random forest classification approach.(Fig. S1A-B). To focus on universally applicable markers for diverse HCC types, we chose 78 markers shared by both cohorts (Fig. S1C) and narrowed it down to 14 markers that consistently showed low levels of methylation (β-value ≤ 0.15 in at least 95% of pan-normal samples) in the TCGA pan-normal datasets. Machine learning methods were used again on the TCGA pan-cancer dataset to focus on regions that were specifically methylated only in HCC but not in other cancer types. Three HCC-specific markers, cg16579555 and cg13204512 within RNF135, and cg02659794 in LDHB, were identified and validated using the test set (one-fifths of the TCGA pan-cancer dataset).

**Fig. 2:**
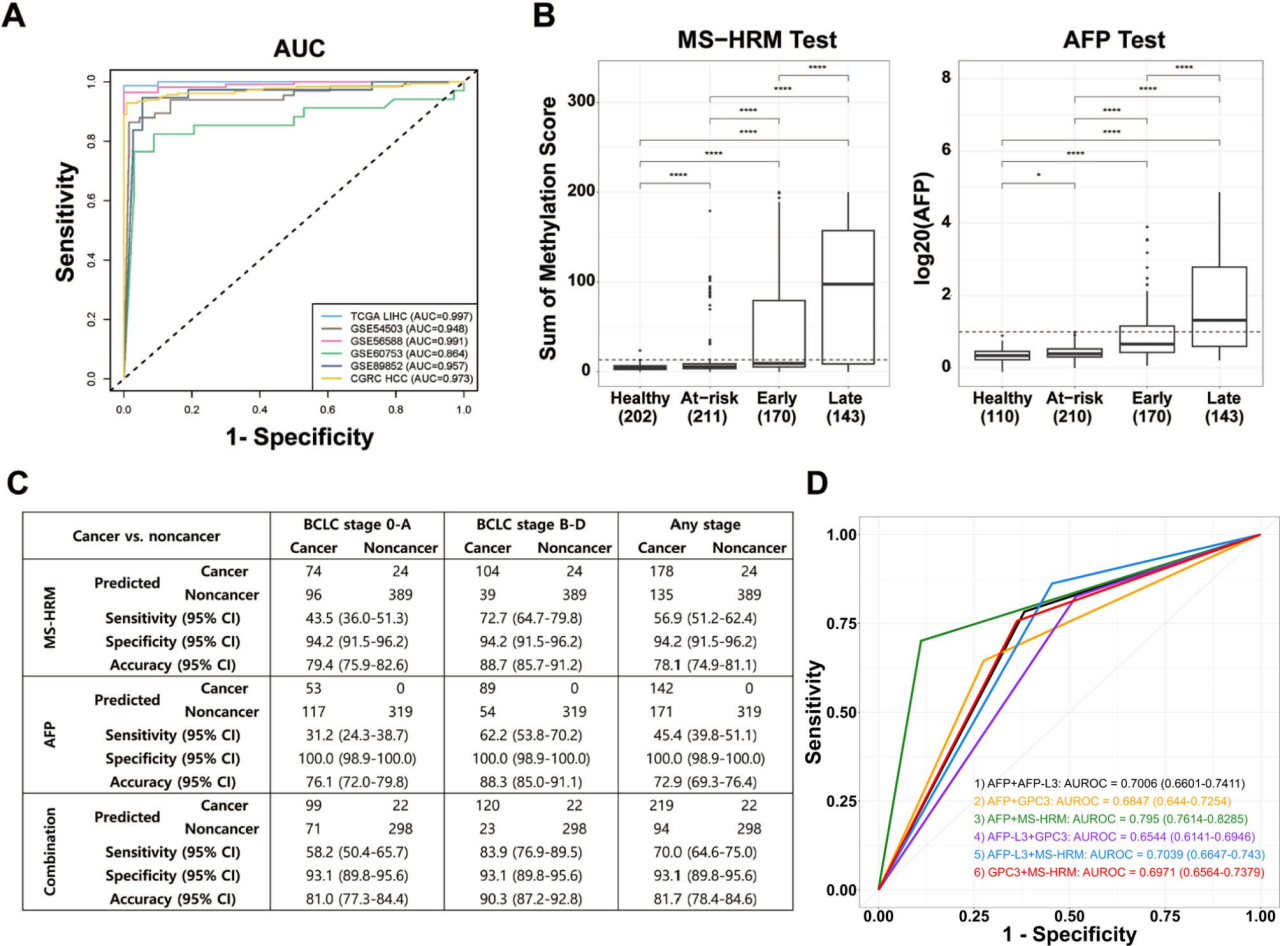
Clinical Performance of Methylation Markers for Detecting HCC. (**A**) AUC analysis of HCC-specific diagnostic markers across four different HCC validation datasets (GSE54503, GSE56588, GSE60753, and GSE89852). The x-axis denotes 1-specificity, the y-axis indicates sensitivity, and the line represents the receiver operating characteristic (ROC) curve for each dataset. (**B**) Boxplot illustrating left) the combined methylation scores for RNF135 and LDHB, as well as right) log20(AFP), across four groups: Healthy, At-risk, Early-stage HCC, and Late-stage HCC. A dotted line marks the 90th quantile of the at-risk group’s sum of methylation score. Log_20_(AFP) set at 1, based on an AFP cutoff of 20 ng/mL, is used as the cutoff value for the AFP tests. (Statistical P values are shown as *, P ≤ 0.05 and ****, P ≤ 0.0001). (**C**) Assay performance table for the MS-HRM and AFP tests. Sensitivity, specificity, and accuracy metric for cancers in the early (BCLC stage 0-A), late (BCLC stage B-D), and any stage are shown. (**D**) ROC curve for the dual-marker combination for 304 HCC patients versus 207 at-risk subjects. The color represents the following combinations: (1) AFP + AFP-L3 (black), (2) AFP + glypican-3 (GPC3) (orange), (3) AFP + MS-HRM (green), (4) AFP-L3 + GPC3 (purple), (5) AFP-L3 + MS-HRM (blue), (6) GPC3 + MS-HRM (red). The x-axis and y-axis represent 1-specificity and sensitivity, respectively

A t-distributed stochastic neighbor embedding (t-SNE) analysis of TCGA data using RNF135 and LDHB DNA methylation patterns produced distinct HCC clusters, confirming the HCC specificity of these methylation markers (Fig. S2). Consistently, these markers showed unmethylated patterns in non-tumoral tissues and other cancer types, while showing significant methylation in HCC tissues (average β-value ≥ 0.3) in the various cancer types in the Gene Expression Omnibus (GEO) database. These findings highlight the high specificity of the RNF135 and LDHB methylation for HCC (Fig. S3). Next, we evaluated the diagnostic performance of these methylation markers in detecting HCC. These candidates performed admirably, with an area under the curve (AUC) of 0.997 (Table S2). This high diagnostic performance was validated in additional four HCC data: GSE54503 (AUC: 94.8%), GSE56588 (AUC: 99.1%), GSE60753 (AUC: 86.4%), and GSE89852 (AUC: 95.7%) (Fig. 2A).

We further investigated the diagnostic precision of RNF135 and LDHB across different developmental stages, etiologies, and racial backgrounds of HCC. Except for dysplastic liver nodules, meta-analysis of RNF135 and LDHB methylation in GEO datasets revealed very low methylation levels in most non-HCC liver tissues, such as liver tissues associated with non-alcoholic fatty liver disease, non-alcoholic steatohepatitis, cirrhosis, hepatocellular adenoma, and intrahepatic bile duct malignancies (intraductal papillary bile duct neoplasms or intrahepatic cholangiocarcinoma) (Fig. S4). Furthermore, the markers’ sensitivity exceeded 93.4% for stages 1 and 2 and approached approximately 100% for stages 3 and 4. Notably, regardless of cause or race, methylation levels of the markers remained consistently high (Fig. S5). These results indicates that methylation changes are heavily accumulated in HCC and begins early in dysplastic stage. These results support their use as universal markers for early detection of HCC, regardless of race or ethnicity.

To explore the potential mechanism underlying the HCC-specific methylation of RNF135 and LDHB, we looked into the connection between the RNF135 and LDHB genes expression and methylation. Both markers showed negative correlation between methylation and expression (Fig. S6). Several studies have shown that hypermethylation of these genes causes their downregulation and is associated with poor survival outcomes RNF135 methylation enhanced HCC cell migration while decreasing immune cell infiltration [11]. Similarly, hypermethylation of LDHB induced a glycolytic shift, boosting cancer cell proliferation and invasion [12].

### MS-HRM analysis for HCC detection: clinical application

Following the identification of the HCC-specific methylation markers, we devised a PCR-based MS-HRM analysis to examine the methylation levels of the markers in clinical samples. MS-HRM analysis generates different melting curve slopes based on methylation levels; higher methylation results in a slower melting curve. After normalizing the melting curves of the tested samples with those of DNA methylation controls, the area under the melting curves (AUMCs) are converted to methylation scores (range from 0 to 100 methylation). To validate the assay’s accuracy, we measured the methylation scores of the markers in tumors and paired normal samples from the liver (58 samples) and non-liver (lung, prostate, colon, stomach, kidney, bladder, and thymus; 5 to 10 samples each). We determined the positive and negative status of each marker based on the 90th quantile of the methylation scores in the normal tissues. Furthermore, we used the ‘sum of methylation score’, which is the sum of these two markers’ methylation levels (RNF135 and LDHB), to classify HCC and non-liver tissue as positive or negative, with an identical cutoff criterion. Both RNF135 and LDHB MS-HRM analysis of tissue samples detected HCC specifically with high precision (RNF135; sensitivity: 72%, specificity: 90%, accuracy: 81% / LDHB; sensitivity: 52%, specificity: 90%, accuracy: 71% / Combination; sensitivity: 72%, specificity: 90%, accuracy: 81%) (Table S3), indicating that this assay can be used to monitor clinical samples for HCC detection.

Following the validation using clinical tissues, we evaluated its accuracy in detecting HCC in blood samples. We examined plasma samples from 202 healthy donors, 211 at-risk individuals, and 313 HCC patients. The baseline characteristics of subjects in the blood sample cohort are described in Table S4. Samples from the healthy and at-risk groups had low methylation scores (RNF135: 3.2%, LDHB: 1.8%), whereas samples from HCC patients had significantly higher methylation scores (RNF135: 6.2%, LDHB: 3.8%, Fig. S7). The methylation scores of RNF135 and LDHB in each sample were added together to form the ‘sum of methylation score’, and the 90th quantile of it from the at-risk groups was used as the cutoff value for HCC detection (Fig. 2B). HCC detection accuracy based on the two methylation marker assay was examined and compared with that of AFP, AFP-L3, and glypican-3 (GPC3) tests. In all HCC stages, our methylation assay detected HCC effectively with better or comparable accuracy than other blood tests (AFP, AFP-L3, and GPC3) (Fig. S8). Interestingly, combining our methylation assay and other blood test yielded a higher sensitivity than either test alone (Fig. 2C), detecting 40–50% additional HCC patients who were negative for both AFP and GPC3 (Fig. S9). These results indicate that our methylation assay has a distinct feature that improves HCC detection sensitivity when used together with other blood tests. The ROC curve analysis supported these findings, demonstrating the combination of AFP and our methylation assay provided the highest level of accuracy (AUC, 0.7950; 95% confidence interval 0.7614–0.8285, Fig. 2D). Indeed, the combined methylation and AFP assay detected 50.6% and 64.5% of very early- and early-stage HCC, respectively, outperforming other available blood tests (Table S5). In addition, we found no significant differences in the assay performance when we examined the sensitivity and specificity of our test results across four etiological categories (HBV, HCV, alcohol, and other etiologies, Table S6).

## Conclusions

In conclusion, we discovered markers with high diagnostic performance across a wide range of HCC types. The datasets used for HCC-specific marker discovery are primarily composed of data from early-stage cancer patients of various racial backgrounds and etiologies. Therefore, the selected markers have significant benefits for early cancer detection and universal application for various types of HCC. Our assay, which is based on two methylation markers, complements the AFP test and outperforms current HCC monitoring tools. A simple PCR-based technique makes the test accessible to at-risk populations. This discovery opens up the possibility of detecting HCC in its most curable stages.

### Electronic supplementary material

Below is the link to the electronic supplementary material.

Supplementary Material 1

Supplementary Material 2

Supplementary Material 3

## Data Availability

The data in this study are available from the corresponding authors upon reasonable request.

## Abbreviations

HCC: Hepatocellular carcinoma
cfDNA: Circulating cell-free DNA
MS-HRM: Methylation-sensitive high-resolution melting
AUC: Area under the curve
AFP: Alpha-fetoprotein
CGRC: Cancer Genome Research Center
TCGA: The Cancer Genome Atlas
HBV: Hepatitis B virus
HCV: Hepatitis C virus
PCA: Principal component analysis
t-SNE: t-distributed stochastic neighbor embedding
RNF135: Ring finger protein 135
LDHB: Lactate dehydrogenase B
GEO: Gene Expression Omnibus
gDNA: genomic DNA
AUMC: Area under the melting curve
