# Supplementary material for: A circulating cell-free DNA methylation signature for the detection of hepatocellular carcinoma
Source: Mol Cancer. 2023 Oct 6;22:164. doi: 10.1186/s12943-023-01872-1 (PMC10557228; doi:10.1186/s12943-023-01872-1)
Supplement: Supplementary file 1 — Supplementary Material 1 [file 12943_2023_1872_MOESM1_ESM.docx]

**Supplementary Materials and Methods**

**Study cohorts**

At the Seoul National University Hospital, two cohorts were gathered for this study, one for HCC tissue study and the other for cfDNA study (Seoul, Republic of Korea). Biopsies were taken from 180 HCC patients who had surgical resection between September 2011 and April 2016 (125 matched normal, 180 tumor). The blood sample cohort included 766 people who were enrolled in an ongoing prospective study to find biomarkers for HCC diagnosis and treatment response [1, 2]. The blood sample cohort was divided into three groups: 1) healthy controls with no history of liver illness or cancer (n = 202), 2) at-risk patients with chronic liver diseases caused by the hepatitis B and C viruses, alcoholic hepatitis, and others (n = 211), and 3) patients with HCC (n = 313). The healthy group was consisted of two groups. Group A included people over the age of 18 who had no history of chronic liver disease, malignancies, or concurrent medication use. Group B included people over the age of 18 who had no clinical or imaging evidence of chronic liver disease or malignancies. Subjects were also excluded from the study if they had (1) abnormal liver biochemistry, (2) tested positive for hepatitis B surface antigen or hepatitis C virus antibodies during the screening blood tests, (3) had elevated liver stiffness values (≥ 7.5 kPa) or controlled attenuation parameters (≥ 260 dB/m) in the screening vibration controlled transient elastography, (4) had a prior history of liver disease, or (5) had a history of any malignancy. Patients with chronic liver diseases caused by hepatitis B and C viruses, alcohol, nonalcoholic fatty liver disease, and other causes were classified as at-risk. Cirrhosis was classified clinically or radiologically using the following criteria: coarse echotexture and nodular liver surface on ultrasound, thrombocytopenia (<150 × 1,000/mm3), or clinical manifestations of portal hypertension (e.g., splenomegaly, ascites, varices). Clinical and biochemical information was obtained from either the registry databases or medical records. HCC was diagnosed based on histological findings or typical imaging features (nodule > 1 cm with arterial hypervascularity and portal/delayed-phase washout) by computed tomography or magnetic resonance imaging (MRI) according to international practice guidelines. The Barcelona Clinic Liver Cancer system was used to define HCC stages [3, 4].

**Sample collection**

Tumor and non-tumor tissue specimens were obtained immediately after liver resection and kept in liquid nitrogen. Plasma was acquired from patients with HCC or chronic liver disease either prospectively, using a PAXgene Blood ccfDNA tube (768115; QIAGEN, Hilden, Germany), or from archived blood samples taken in an EDTA tube. 50 ml of blood was taken prospectively from healthy controls using an AlphaLiquid^TM^ (IMBdx, Republic of Korea) or EDTA tube (BD Vacutainer, Franklin Lakes, NJ, USA). The plasma supernatant was transferred to a 15-ml tube after blood was collected in AlphaLiquid^TM^ or PAXgene ccfDNA tubes and centrifuged at 1,900 g (15-25 °C) for 15 minutes. The obtained plasma was centrifuged again for 15 minutes at 1,900 g (15-25 °C), and the supernatant was collected to get a sample as clean of debris as feasible. Plasma was extracted from blood samples obtained in an EDTA tube using conventional methods. All samples were kept at 80 °C until they were analyzed. For all samples collected, written informed consent was obtained.

**Methylation data used for HCC-specific marker discovery.**

To identify changes in DNA methylation, samples from 180 patients and matched non-tumor tissues from 125 individuals were analyzed with the 850K microarray platform (described in greater detail below). The cohort was referred to as the CGRC HCC cohort. In addition, data generated by the 450K microarray from TCGA cohort samples (379 tumor and 50 non-tumor tissues) were obtained for the 'TCGA HCC' cohort. To identify HCC-specific diagnostic markers, pan-cancer methylome datasets of human tissues were obtained from the TCGA database (684 normal samples and 7,296 tumor samples). Four independent HCC cohorts were used to validate these probes: GSE54503 (normal, 66; tumor, 66), GSE56588 (normal, 10; tumor, 224), GSE60753 (normal, 34; tumor, 34), and GSE89852 (normal, 37; tumor, 37). The datasets utilized for validating liver cancer-specific markers are listed in Supplementary Table S7.

**Methylation microarrays**

The Infinium Human Methylation EPIC 850K BeadChip (Illumina, San Diego, CA, USA) was employed to generate DNA methylation data for genomic DNA (gDNA) samples from the CGRC cohort. This chip assesses methylation levels at over 850,000 CpG sites following the manufacturer's standard protocol. Subsequent to data acquisition, the raw fluorescence signal intensity was processed to derive methylation β-values, and these values were mapped to the hg19 reference genome. For all steps related to quality control, preprocessing, and normalization of the raw data, we utilized the "minfi" R package (version 3.4.3) [5]. The "minfi" package is a versatile and comprehensive Bioconductor tool designed specifically for the analysis of Infinium DNA methylation microarrays. The package enables robust and reliable analysis of DNA methylation data by facilitating a variety of operations such as quality assessment, data preprocessing, and normalization. DNA methylation data collected by the Infinium Methylation450 Bead Chip technology were retrieved from the TCGA database for the TCGA cohort and GEO database. The methylation β-values varied from 0 (unmethylated) to 1 (completely methylated).

**Clustering and visualization**

The 'prcomp' package in R (v.3.6.2) for PCA and the 'Rtsne' package in R for t-SNE were used for the clustering analysis. Gene Cluster v.3.0 was used to do hierarchical clustering, and Java TreeView v.1.1.6 was used to display the outcomes.

**Random forest analysis**

The machine learning model was constructed using the 'randomForest' package in R (v. 4.6.14). For this approach, the dataset was randomly partitioned into two subsets: training (comprising 80% of the data) and testing (making up the remaining 20%). The training set was used to construct the machine learning model, while the testing set was used to evaluate its predictive accuracy. The model's performance was subsequently validated through a cross-validation procedure using an independent liver cancer cohort. We employed the AUC as our main evaluative metric for gauging the model's diagnostic performance, which enabled us to assess both its sensitivity and specificity.

**Control DNA, gDNA, and cfDNA extraction and bisulfite conversion**

The EpiTect PCR Control DNA Set (QIAGEN, 59695) containing fully methylated DNA, unmethylated DNA, and gDNA was used as control DNA. We extracted gDNA of fresh frozen tissues from CGRC cohort using the QIAamp DNA Mini Kit (QIAGEN, 51304) according to manufacturer’s instructions. We extracted cfDNA from approximately 2-5 mL plasma using the MagListo^TM^ cfDNA Extraction Kit (Bioneer, K-3619), according to the manufacturer’s instructions. Isolated gDNA and cfDNA were bisulfite converted using the EZ DNA Methylation-Lightning Kit (D5030; Zymo Research, Irvine, CA, USA), according to the manufacturer’s instructions.

**MS-HRM analysis**

MS-HRM analysis was used to evaluate two differentially methylated areas for the diagnosis of HCC. To prevent unmethylated template bias amplification, primers for MS-HRM were constructed with 1-2 CpGs at the 5′ end. The CFX Connect Real-Time PCR Detection System (Bio-Rad, Hercules, CA, USA) was used for PCR amplification and HRM analysis. For each marker, PCR reaction mixtures (25 uL total volume) were produced with the appropriate primer concentration (RNF135, common forward 0.4 μM, common reverse 0.4 μM; LDHB, methylated forward 0.4 μM, unmethylated forward 0.2 μM, common reverse 0.4 μM). The CFX Connect cycling protocol for HRM analysis is as follows: initial denaturation at 95 °C for 5 min; then 21 cycles at 95 °C denaturation for 20 s, annealing and extension at 69 °C for 40 s (decreasing temperature by ‒0.3 °C per cycle, touch-down PCR); and then 50 cycles at 95 °C denaturation for 20 s, and annealing and extension at 63 °C for 40 s; followed by a final extension step at 63 °C for 5 min. HRM analysis was performed at temperatures ranging from 60 to 95 °C, with a ramp rate of 0.2 °C per 10 s and a fluorescence acquisition setup. The EpiTect PCR Control DNA Set (QIAGEN, 59695) containing fully methylated DNA, unmethylated DNA, and gDNA was used as control DNA.

Raw experimental data from each MS-HRM experiment were processed as follows to obtain a single concise methylation value. We first created an exponential background model and subtracted it from the raw relative fluorescence units’ curve [6]. The resulting curve was min-max normalized into a DNA melting curve with values ranging from 0 to 1. The area under the DNA melting curve (AUMC) based on cubic spline interpolation was subsequently described as AUMC, which has a monotonic direct relationship with the proportions of methylated cytosines inside the amplicon. We discovered different AUMC values as a result of minor changes in ion concentrations in the PCR mixture and discrepancies in qPCR machines. To correct for this variance, we used the temperature-shift approach to change the melting curve depending on a specified temperature, thereby reducing the experimental parameters that influenced HRM results. Min-max normalization of the temperature-shifted AUMC values of each sample was conducted to derive the methylation score based on the temperature-shifted AUMC values obtained from the 100% methylated control and the unmethylated control, as follows: [7]

$$\boldsymbol{Methylation Score=}\frac{\boldsymbol{AUMC}_{\boldsymbol{Sample}}\boldsymbol{-}\boldsymbol{AUMC}_{\boldsymbol{Unmet. Ctrl.}}}{\boldsymbol{AUMC}_{\boldsymbol{Met. Ctrl}}\boldsymbol{-}\boldsymbol{AUMC}_{\boldsymbol{Unmet. Ctrl.}}}$$

$$\left\{ \begin{aligned} \boldsymbol{Methylation Score < 0, 0} \\ \boldsymbol{Methylation Score \geq100, 100} \end{aligned} \right.$$

**Selected reaction monitoring-mass spectrometry analysis**

Target peptides of AFP, AFP-L3 and glypican-3 were quantified on an Agilent 6495C triple quadrupole (QQQ) mass spectrometer (Agilent) with a Jetstream electrospray source that was equipped with a 1290 Infinity HPLC system (Agilent). Ten microliters of tryptic peptides were injected into a guard column (2.1 mm id × 30 mm, 1.8μm particle size, Agilent) for online desalting and then passed through to a reverse-phase analytical column (2.1 mm id × 150 mm id, 1.8μm particle size, Agilent) that was maintained at 40°C. The total run time for the liquid chromatography (LC) step was 70 min. Mobile phases A (water 0.1% (v/v) formic acid) and B (acetonitrile 0.1% (v/v) formic acid) were used to create a binary gradient of 3 to 35%acetonitrile/0.1% formic acid through the column for 10 min at 400μL/min to separate the peptides. The column was equilibrated for the next run by ramping the gradient to 70% B for 2 min and 3% B for 3 min. Mass Hunter (vB06.01, Agilent Technologies) was used to establish a selected reaction monitoring-mass spectrometry (SRM−MS) scheduling method and control the LC−MS system for data acquisition. The ion spray capillary voltage and nozzle voltage were 2500 and 2000 V, respectively. The temperature and flow rate of the gas were set to 250°Cat15 L/min for the drying gas and 350°C at 12 L/min for the sheath gas. The voltage of the cell accelerator was adjusted to 5V, the fragment voltage was 380 V, and the delta electron multiplier voltage (EMV) was 200 V. A total of three batches were sequentially analyzed in order of preparation, whereas samples in each batch were analyzed in random sequence. All raw datafiles from the SRM−MS were imported into and aligned by Skyline (MacCoss Lab, University of Washington) for quantitative analysis. The AFP-L3 measurement values were calculated as a percentage by dividing AFP-L3 intensity by the sum of AFP and AFP-L3 intensity. The optimal cut-off of each marker was determined by the Youden index.

**Supplementary References**

1. Kim, D.J., et al., *Comprehensive Metabolomic Search for Biomarkers to Differentiate Early Stage Hepatocellular Carcinoma from Cirrhosis.* Cancers (Basel), 2019. **11**(10).

2. Kim, H., et al., *Prediction of Response to Sorafenib in Hepatocellular Carcinoma: A Putative Marker Panel by Multiple Reaction Monitoring-Mass Spectrometry (MRM-MS).* Molecular Cell Proteomics, 2017. **16**(7): p. 1312-1323.

3. Reig, M., et al., *BCLC strategy for prognosis prediction and treatment recommendation: The 2022 update.* Journal of hepatology, 2022. **76**(3): p. 681-693.

4. Marrero, J.A., et al., *Diagnosis, S taging, and M anagement of H epatocellular C arcinoma: 2018 P ractice G uidance by the A merican A ssociation for the S tudy of L iver D iseases.* Hepatology, 2018. **68**(2): p. 723-750.

5. Aryee, M.J., et al., *Minfi: a flexible and comprehensive Bioconductor package for the analysis of Infinium DNA methylation microarrays.* Bioinformatics, 2014. **30**(10): p. 1363-9.

6. Palais, R. and C.T. Wittwer, *Mathematical algorithms for high-resolution DNA melting analysis.* Methods Enzymology, 2009. **454**: p. 323-43.

7. Raschka, S., *About feature scaling and normalization and the effect of standardization for machine learning algorithms.* Polar Political Legal Anthropology Rev, 2014. **30**(1): p. 67-89.
