# Supplementary material for: A circulating cell-free DNA methylation signature for the detection of hepatocellular carcinoma
Source: Mol Cancer. 2023 Oct 6;22:164. doi: 10.1186/s12943-023-01872-1 (PMC10557228; doi:10.1186/s12943-023-01872-1)
Supplement: Supplementary file 2 — Supplementary Material 2 [file 12943_2023_1872_MOESM2_ESM.docx]

**Supplementary Tables**

**Supplementary Table S1.** Characteristics of the two discovery cohorts.

|  | **CGRC HCC  (*n* = 180)** | **TCGA LIHC  (*n* = 379)** |
| --- | --- | --- |
| **Sex, *n* (%)** |  |  |
| Male | 138 (76.7) | 254 (67.0) |
| Female | 42 (23.3) | 122 (32.2) |
| NA | 0 (0) | 3 (0.8) |
| **Age** |  |  |
| ≤65 | 139 (77.2) | 235 (62.0) |
| >65 | 41 (22.8) | 141 (37.2) |
| NA | 0 (0) | 3 (0.8) |
| **Race** |  |  |
| White | 0 (0) | 187 (49.3) |
| Asian | 178 (98.9) | 160 (42.2) |
| Black | 0 (0) | 17 (4.5) |
| Other | 0 (0) | 12 (3.2) |
| NA | 2 (1.1) | 3 (0.8) |
| **HBsAg** |  |  |
| Positive | 154 (85.6) | 107 (28.2) |
| Negative | 26 (14.4) | 269 (71.0) |
| NA | 0 (0) | 3 (0.8) |
| **Anti-HCV** |  |  |
| Positive | 15 (8.3) | 56 (14.8) |
| Negative | 165 (91.7) | 320 (84.4) |
| NA | 0 (0) | 3 (0.8) |
| **Child-Pugh grade** |  |  |
| A | 180 (100) | 105 (27.7) |
| B | 0 (0) | 10 (2.6) |
| NA | 0 (0) | 264 (69.7) |
| **AJCC Pathologic Primary Tumor (pT)^a^** |  |  |
| T1,2 | 153 (85.0) | 279 (73.6) |
| T3,4 | 13 (7.2) | 94 (24.8) |
| NA | 0 (0) | 6 (1.6) |
| **AFP (ng/ml)** |  |  |
| ≤100 | 132 (73.3) | 196 (51.7) |
| >100 | 48 (26.7) | 87 (23.0) |
| NA | 0 (0) | 96 (25.3) |
| **PIVCA-II (mAU/mL)** |  |  |
| ≤40 | 51 (28.3) | 0 (0) |
| >40 | 129 (71.7) | 0 (0) |
| NA | 0 (0) | 379 (100) |

^a^The 7^th^ edition of the AJCC cancer staging system for HCC was used.

AFP, alpha-fetoprotein; AJCC, American Joint Committee on Cancer; CGRC, Cancer Genome Research Center; HBsAg, hepatitis B surface antigen; HCC, hepatocellular carcinoma; HCV, hepatitis C virus; LIHC, liver hepatocellular carcinoma; NA, not assessable; PIVKA-II, prothrombin induced by vitamin K absence or antagonist-II; TCGA, The Cancer Genome Atlas.

**Supplementary Table S2.** Confusion tables for the diagnostic random forest model.

|  |  | **Actual** | | | |
| --- | --- | --- | --- | --- | --- |
|  |  | **Training set** | | **Test set** | |
|  |  | LIHC_T | Other_T | LIHC_T | Other_T |
| **Predict** | LIHC_T | 246 | 57 | 66 | 1 |
|  | **Other_T** | 7 | 5829 | 10 | 1459 |
| **Sensitivity (%)** | | 97.2 | | 86.8 | |
| **Specificity (%)** | | 99.0 | | 99.9 | |
| **Accuracy (%)** | | 99.0 | | 99.3 | |

Confusion matrix of our random forest model in training (left) and testing (right) datasets.

LIHC, liver hepatocellular carcinoma; T, tumor

**Supplementary Table S3.** MS-HRM confusion table for HCC and other tumor types.

| **Type** | | **RNF135** | | | **LDHB** | | | **Sum of Methylation Score** | | |
| --- | --- | --- | --- | --- | --- | --- | --- | --- | --- | --- |
|  |  | **Positive** | **Negative** | **Se. / Sp. / Acc.** | **Positive** | **Negative** | **Se. / Sp. / Acc.** | **Positive** | **Negative** | **Se. / Sp. / Acc.** |
| **LIHC** | **Tumor** | 42 | 16 | 72.4% / 89.7% / 81.0% | 30 | 28 | 51.7% / 89.7% / 70.7% | 42 | 16 | 72.4% / 89.7% / 81.0% |
|  | **Normal** | 6 | 52 |  | 6 | 52 |  | 6 | 52 |  |
| **LUAD** | **Tumor** | 0 | 9 | - / 100.0% / - | 1 | 8 | - / 88.2% / - | 1 | 8 | - / 88.2 / - |
|  | **Normal** | 0 | 8 |  | 1 | 7 |  | 1 | 7 |  |
| **PRAD** | **Tumor** | 1 | 7 | - / 93.8% / - | 0 | 8 | - / 100.0% / - | 1 | 7 | - / 93.8% / - |
|  | **Normal** | 0 | 8 |  | 0 | 8 |  | 0 | 8 |  |
| **COAD** | **Tumor** | 0 | 10 | - / 100.0% / - | 0 | 10 | - / 100.0% / - | 0 | 10 | - / 100.0% / - |
|  | **Normal** | 0 | 10 |  | 0 | 10 |  | 0 | 10 |  |
| **STAD** | **Tumor** | 0 | 8 | - / 94.1% / - | 2 | 6 | - / 88.2% / - | 2 | 6 | - / 88.2% / - |
|  | **Normal** | 1 | 8 |  | 0 | 9 |  | 0 | 9 |  |
| **KIRP** | **Tumor** | 0 | 5 | - / 100.0% / - | 0 | 5 | - / 92.9% / - | 1 | 4 | - / 92.9% / - |
|  | **Normal** | 0 | 9 |  | 1 | 8 |  | 0 | 9 |  |
| **KIRC** | **Tumor** | 1 | 9 | - / 94.1% / - | 0 | 7 | - / 100.0% / - | 1 | 9 | - / 94.1% / - |
|  | **Normal** | 0 | 7 |  | 0 | 10 |  | 0 | 7 |  |
| **BLCA** | **Tumor** | 1 | 8 | - / 89.5% / - | 0 | 9 | - / 94.7% / - | 0 | 9 | - / 94.7 / - |
|  | **Normal** | 1 | 9 |  | 1 | 9 |  | 1 | 9 |  |
| **THYM** | **Tumor** | 0 | 6 | - / 91.7% / - | 0 | 6 | - / 100.0% / - | 0 | 6 | - / 100.0% / - |
|  | **Normal** | 1 | 5 |  | 0 | 6 |  | 0 | 6 |  |

LIHC, Liver Hepatocellular Carcinoma; LUAD, Lung Adenocarcinoma; PRAD, Prostate adenocarcinoma; COAD, Colon adenocarcinoma; STAD, Stomach adenocarcinoma; KIRP, Kidney renal papillary cell carcinoma; KIRC, Kidney renal clear cell carcinoma; BLCA, Bladder Urothelial Carcinoma; THYM, Thymoma; Se., Sensitivity; Sp., Specificity; Acc., Accuracy

**Supplementary Table S4.** Baseline characteristics of the blood sample cohort.

|  | **Healthy**  **(*n* = 202)** | **At-risk**  **(*n* = 211)** | **HCC**  **(*n*** = **313)** |
| --- | --- | --- | --- |
| **Sample type, n (%)** |  |  |  |
| Archival Sample | 0 (0.0%) | 210 (99.5%) | 152 (48.6%) |
| Prospective Sample | 202 (100.0%) | 1 (0.5%) | 161 (51.4%) |
| **Gender** |  |  |  |
| Male | 118 (58.4%) | 107 (50.7%) | 247 (78.9%) |
| Female | 84 (41.6%) | 104 (49.3%) | 66 (21.1%) |
| **Age** |  |  |  |
| <30 | 20 (9.9%) | 3 (1.4%) | 0 (0.0%) |
| 30≤ *n* <40 | 37 (18.3%) | 22 (10.4%) | 2 (0.6%) |
| 40≤ *n* <50 | 54 (26.7%) | 46 (21.8%) | 29 (9.3%) |
| 50≤ *n* <60 | 42 (20.8%) | 71 (33.6%) | 91 (29.1%) |
| 60≤ *n* <70 | 45 (22.3%) | 45 (21.3%) | 118 (37.7%) |
| ≤70 | 4 (2.0%) | 24 (11.3%) | 73 (23.3%) |
| **Etiology of the underlying chronic liver disease** |  |  |  |
| HBV | 0 (0.0%) | 166 (78.7%) | 243 (77.6%) |
| HCV | 0 (0.0%) | 15 (7.1%) | 33 (10.5%) |
| Alcohol | 0 (0.0%) | 27 (12.8%) | 20 (6.4%) |
| Others | 0 (0.0%) | 3 (1.4%) | 17 (5.4%) |
| **Size of the largest tumor (cm)** |  |  |  |
| <3 | - | - | 149 (47.6%) |
| ≥3 | - | - | 164 (52.4%) |
| **BCLC stage** |  |  |  |
| 0, A | - | - | 170 (54.3%) |
| B, C, D | - | - | 143 (45.7%) |
| **AFP (ng/ml)** |  |  |  |
| <20 | 110 (54.5%) | 210 (99.5%) | 171 (54.6%) |
| ≥20 | - | - | 142 (45.4%) |
| NA | 92 (45.5%) | 1 (0.5%) | - |

HBV, hepatitis B virus; HCV, hepatitis C virus; BCLC, Barcelona Clinic Liver Cancer; AFP, alpha-fetoprotein; HCC, hepatocellular carcinoma.

**Supplementary Table S5.** Clinical performance of the MS-HRM and AFP assays stratified by BCLC stage.

| **BCLC Stage** | | **0** | **A** | **B** | **C** | **D** | **Any** |
| --- | --- | --- | --- | --- | --- | --- | --- |
| **AFP** | **Pos.** | 22 | 31 | 13 | 74 | 2 | 142 |
|  | **Neg.** | 55 | 62 | 21 | 29 | 4 | 171 |
|  | **Total** | 77 | 93 | 34 | 103 | 6 | 313 |
|  | **Se.** | 28.6% | 33.3% | 38.2% | 71.8% | 33.3% | 45.4% |
| **`** | | | | | | | |
| **MS-HRM** | **Pos.** | 27 | 47 | 21 | 79 | 4 | 178 |
|  | **Neg.** | 50 | 46 | 13 | 24 | 2 | 135 |
|  | **Total** | 77 | 93 | 34 | 103 | 6 | 313 |
|  | **Se.** | 35.1% | 50.5% | 61.8% | 76.7% | 66.7% | 56.9% |
|  | | | | | | | |
| **Combination^*^** | **Pos.** | 39 | 60 | 23 | 93 | 4 | 219 |
|  | **Neg.** | 38 | 33 | 11 | 10 | 2 | 94 |
|  | **Total** | 77 | 93 | 34 | 103 | 6 | 313 |
|  | **Se.** | 50.6% | 64.5% | 67.6% | 90.3% | 66.7% | 70.0% |

BCLC, Barcelona Clinic Liver Cancer; Pos., positive; Neg., negative; Se., sensitivity

^*^ If either the AFP test or the MS-HRM test was positive, the combination result was considered positive

**Supplementary Table S6.** Clinical performance of the MS-HRM assay stratified by etiology.

| **Etiology** | **BCLC stage 0-A** | | | **BCLC stage B-D** | | | **Any stage** | | | **At-risk** | | |
| --- | --- | --- | --- | --- | --- | --- | --- | --- | --- | --- | --- | --- |
|  | **Pos.** | **Neg.** | **Se.** | **Pos.** | **Neg.** | **Se.** | **Pos.** | **Neg.** | **Se.** | **Pos.** | **Neg.** | **Sp.** |
| **HBV** | 52 | 81 | 39.1 | 81 | 29 | 73.6 | 133 | 110 | 54.7 | 10 | 156 | 94.0 |
| **HCV** | 13 | 10 | 56.5 | 8 | 2 | 80.0 | 21 | 12 | 63.6 | 4 | 11 | 73.3 |
| **Alcohol** | 6 | 2 | 75.0 | 6 | 6 | 50.0 | 12 | 8 | 60. | 8 | 19 | 70.4 |
| **Other** | 3 | 3 | 50.0 | 9 | 2 | 81.8 | 12 | 5 | 70.6 | 1 | 2 | 66.7 |
| **Total** | 74 | 96 | 43.5 | 104 | 39 | 72.7 | 178 | 135 | 56.9 | 23 | 188 | 89.1 |

Pos., Positive; Neg., Negative; Se., Sensitivity; Sp., Specificity

**Supplementary Table S7.** Datasets utilized to validate liver cancer-specific markers.

| **Type** | | **N** | **T** |
| --- | --- | --- | --- |
| **Bladder** | BLCA | 21 | 413 |
|  | GSE52955 | 5 | 25 |
| **Breast** | BRCA | 97 | 772 |
|  | GSE39451 | - | 20 |
|  | GSE52865 | 17 | 40 |
|  | GSE60185 | 46 | 239 |
| **Cervix** | CESC | 3 | 309 |
|  | GSE46306 | 20 | 6 |
| **Colorectal** | CGRC_CRC | 351 | 775 |
|  | COAD | 38 | 297 |
|  | GSE39958 | - | 45 |
|  | GSE42752 | 19 | 44 |
|  | GSE48684 | 17 | 63 |
|  | READ | 7 | 99 |
| **Esophagus** | ESCA | 16 | 186 |
|  | GSE52826 | 8 | 4 |
| **Glioblastoma** | GBM | 1 | 153 |
|  | GSE36278 | 6 | 136 |
|  | GSE58298 | - | 40 |
|  | GSE60274 | 5 | 72 |
| **Liver** | CGRC_HCC | 127 | 182 |
|  | GSE54503 | 41 | 66 |
|  | GSE56588 | 10 | 224 |
|  | GSE60753 | 34 | 34 |
|  | GSE89852 | 37 | 37 |
|  | LIHC | 50 | 379 |
| **Bile duct** | CHOL | 9 | 36 |
| **Head and Neck** | GSE38266 | - | 41 |
|  | GSE40005 | 12 | 12 |
|  | HNSC | 50 | 530 |
| **Kidney** | GSE50874 | 85 | - |
|  | GSE61441 | 46 | 46 |
|  | KIRC | 160 | 320 |
|  | KIRP | 45 | 276 |
| **Lung** | CGRC_lung | - | 42 |
|  | GSE39279 | - | 444 |
|  | GSE52401 | 244 | - |
|  | GSE56044 | 12 | 124 |
|  | LUAD | 32 | 460 |
|  | LUSC | 42 | 370 |
| **Pancreas** | GSE49149 | 29 | 167 |
|  | PAAD | 10 | 185 |
| **Paraganglion** | GSE43293 | 8 | 16 |
|  | PCPG | 3 | 184 |
| **Prostate** | GSE47915 | 4 | 4 |
|  | GSE55598 | 16 | 16 |
|  | PRAD | 50 | 499 |
| **Sarcoma** | SARC | 4 | 265 |
| **Skin** | SKCM | 2 | 473 |
| **Stomach** | GSE34387 | 7 | 69 |
|  | STAD | 2 | 395 |
| **Thymus** | GSE55111 | 11 | - |
|  | THCA | 56 | 517 |
|  | THYM | 2 | 124 |
| **Uterine** | GSE45187 | 3 | 6 |
|  | UCEC | 34 | 432 |
| **Blood** | GSE35069 | 6 | - |
|  | GSE41169 | 33 | - |
|  | GSE54570 | 6 | - |
|  | GSE64495 | 62 | - |
|  | KNIH | 400 | - |

BLCA, Bladder Urothelial Carcinoma; BRCA, Breast Invasive Carcinoma; CESC, Cervical Squamous Cell Carcinoma and Endocervical Adenocarcinoma; COAD, Colon Adenocarcinoma; READ, Rectum Adenocarcinoma; ESCA, Esophageal Carcinoma; GBM, Glioblastoma Multiforme; LIHC, Liver Hepatocellular Carcinoma; CHOL, Cholangiocarcinoma; HNSC, Head and Neck Squamous Cell Carcinoma; KIRC, Kidney Renal Clear Cell Carcinoma; KIRP, Kidney Renal Papillary Cell Carcinoma; LUAD, Lung Adenocarcinoma; LUSC, Lung Squamous Cell Carcinoma; PAAD, Pancreatic Adenocarcinoma; PCPG, Pheochromocytoma and Paraganglioma; PRAD, Prostate Adenocarcinoma; SARC, Sarcoma; SKCM, Skin Cutaneous Melanoma; STAD, Stomach Adenocarcinoma; THCA, Thyroid Carcinoma; THYM, Thymoma; UCEC, Uterine Corpus Endometrial Carcinoma: N: normal; T: tumor.
