# Supplementary material for: A circulating cell-free DNA methylation signature for the detection of hepatocellular carcinoma
Source: Mol Cancer. 2023 Oct 6;22:164. doi: 10.1186/s12943-023-01872-1 (PMC10557228; doi:10.1186/s12943-023-01872-1)
Supplement: Supplementary file 3 — Supplementary Material 3 [file 12943_2023_1872_MOESM3_ESM.docx]

**Supplementary figures**

**Supplementary Figure 1: HCC methylation markers in CGRC and TCGA cohorts.**

(A-B) The variable importance plot derived from the random forest model for the CGRC HCC (A) and TCGA LIHC (B) cohorts. The x-axis denotes the mean decrease in accuracy, the y-axis signifies the mean decrease in the Gini score, and the blue dots represent individual probes.

(C) A scatter plot showcasing the top 100 HCC diagnostic candidates from each platform (CGRC HCC and TCGA LIHC). The X-axis indicates the importance rank of the probe in the TCGA cohort, while the Y-axis shows the importance rank of the probe in the CGRC cohort. Red circles highlight probes that are significantly prominent on both platforms.

**
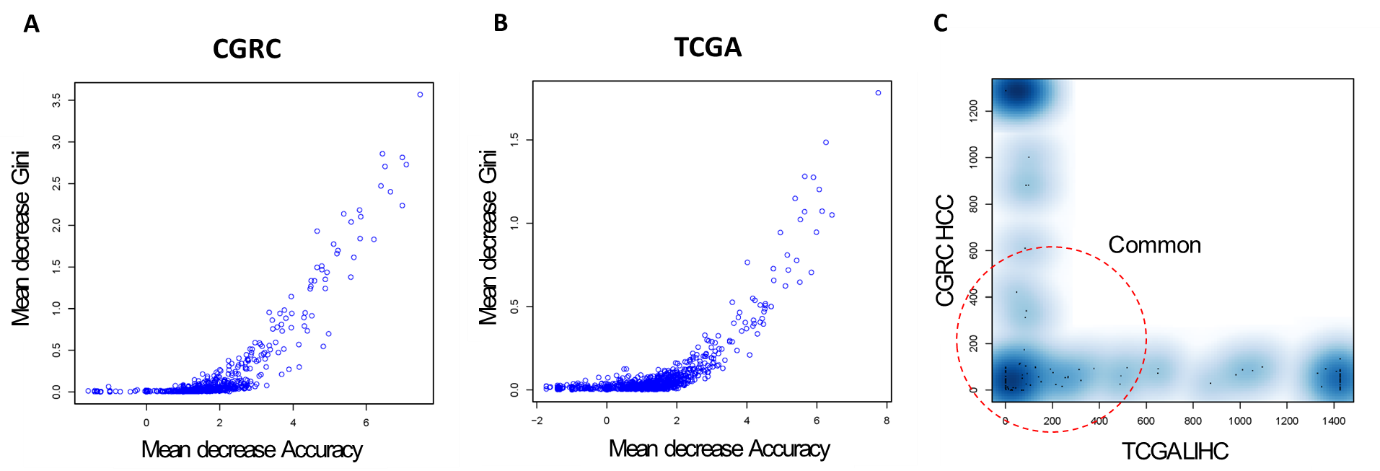
**

**Supplementary Figure 2: Validation of HCC-specific methylation markers.**

(A) A t-SNE plot illustrating the methylation distribution of HCC-specific diagnostic candidates across different sample groups: whole blood (blue dot; n = 507), pan-normal samples from TCGA (green dot; n = 684), and HCC samples from CGRC (pink dot; n = 180) and TCGA (purple dot; n = 379).

(B) A t-SNE plot displaying the methylation distribution of HCC-specific diagnostic candidates in HCC samples from CGRC (pink dot; n = 180) and TCGA (purple dot; n = 379) in contrast to other cancer types (black dot; n = 7,296).


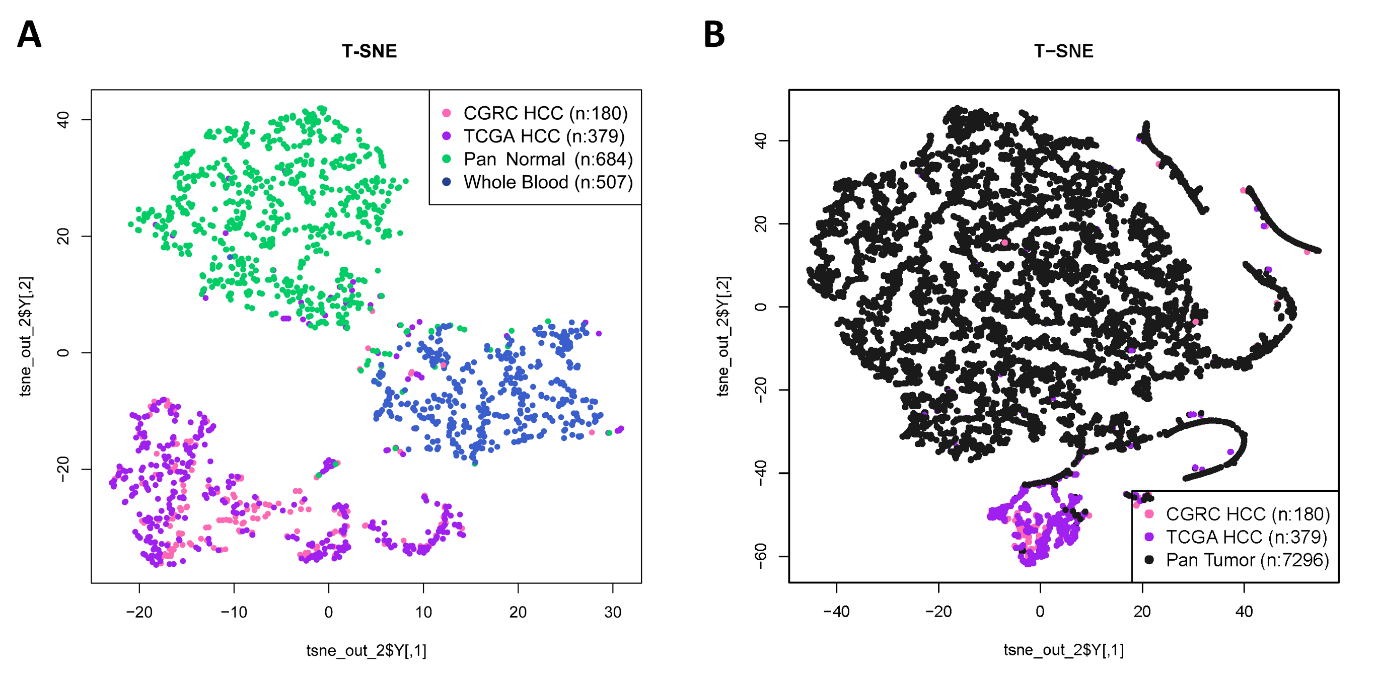


**Supplementary Figure 3: Specificity of HCC methylation markers.**

(A-B) Methylation distribution of HCC-specific **methylation** markers across various tissue types from the GEO database. The left panel displays normal tissue, and the right panel shows tumor tissue. The x-axis lists different tissue types, and the y-axis shows methylation β-values. β-values span from 0 to 1, with green boxes indicating normal samples and pink boxes indicating HCC samples. The central line in the box plot marks the mean values for each dataset. The list of samples used in the box plots is shown in Table S7.


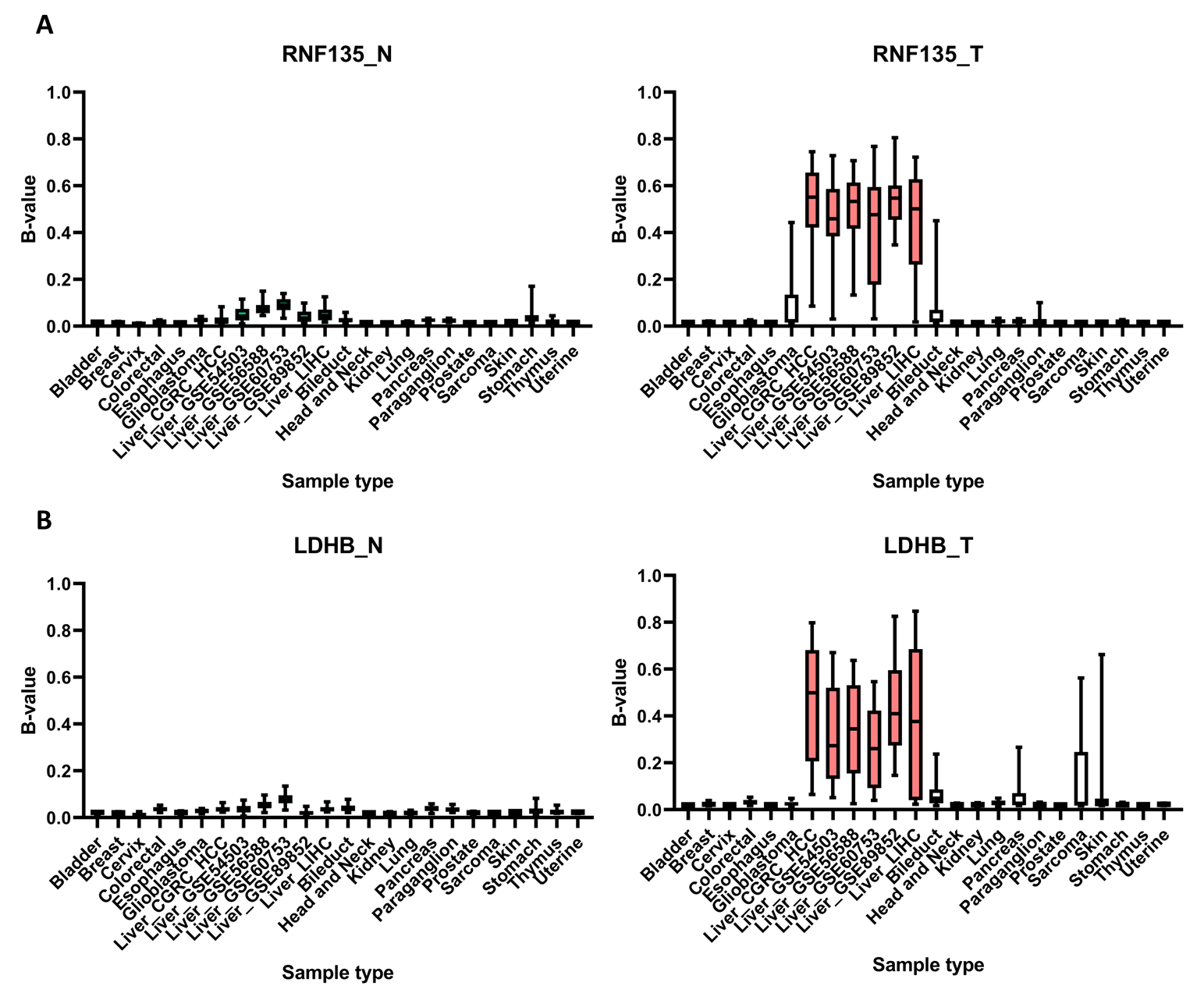


**Supplementary Figure 4: Detection accuracy of HCC-specific methylation markers.**

(A) A box plot showing the methylation distribution of HCC-specific methylation markers across various hepatobiliary cancers, including CCA and HCC. β-values range from 0 to 1, with pink boxes indicating HCC samples.

(B) A box plot illustrating the methylation distribution of HCC-specific methylation markers in liver diseases and conditions leading to hepatocarcinogenesis, such as NAFLD, NASH, cirrhosis, adenoma, and dysplastic nodules. β-values span from 0 to 1, with the pink box indicaing HCC samples.

The following data sets were used in the analysis: Bile Duct Normal (GSE156299 (n=50), GSE89803 (n=4)), IPNB (GSE156299 (n=41)), ITPN (GSE156299 (n=10)), ITPN-P (GSE156299 (n=9)), Extrahepatic Cholangiocarcinoma (eCCA) (GSE60446 (n=6)), Intrahepatic Cholangiocarcinoma (iCCA) (GSE156299 (n=9), GSE60446 (n=6)), Distal Intrahepatic Cholangiocarcinoma (dCCA) (GSE156299 (n=10)), Perihilar Intrahepatic Cholangiocarcinoma (pCCA) (GSE156299 (n=8)), Intrahepatic Cholangiocarcinoma (CCA) (GSE32079 (n=31), GSE49656 (n=32), GSE89803 (n=138)), Hepatocellular Carcinoma (HCC) (GSE146286 (n=8), GSE99036 (n=15), CGRC HCC (n=180), GSE54503 (n=66), GSE56588 (n=224), GSE60753 (n=34), GSE89852 (n=37), LIHC (n=379)), HepG2 (GSE60753 (n=8)), Liver Normal (GSE52731 (n=70), GSE43091 (n=4), CGRC HCC (n=125), GSE54503 (n=41), GSE56588 (n=10), GSE60753 (n=34), GSE89852 (n=37), LIHC (n=50)), NAFLD (GSE61258 (n=14), GSE49542 (n=59)), NASH (GSE61258 (n=7), GSE48325 (n=15)), Cirrhosis (GSE129374 (n=22), GSE157973 (n=130), GSE146286 (n=7), GSE99036 (n=6), GSE60753 (n=77)), Adenoma (GSE43091 (n=50)), Dysplastic Liver (GSE157973 (n=8)), Low-Grade Dysplastic Nodule (LGDN) (GSE146286 (n=3)), High-Grade Dysplastic Nodule (HGDN) (GSE146286 (n=10), GSE99036 (n=11))


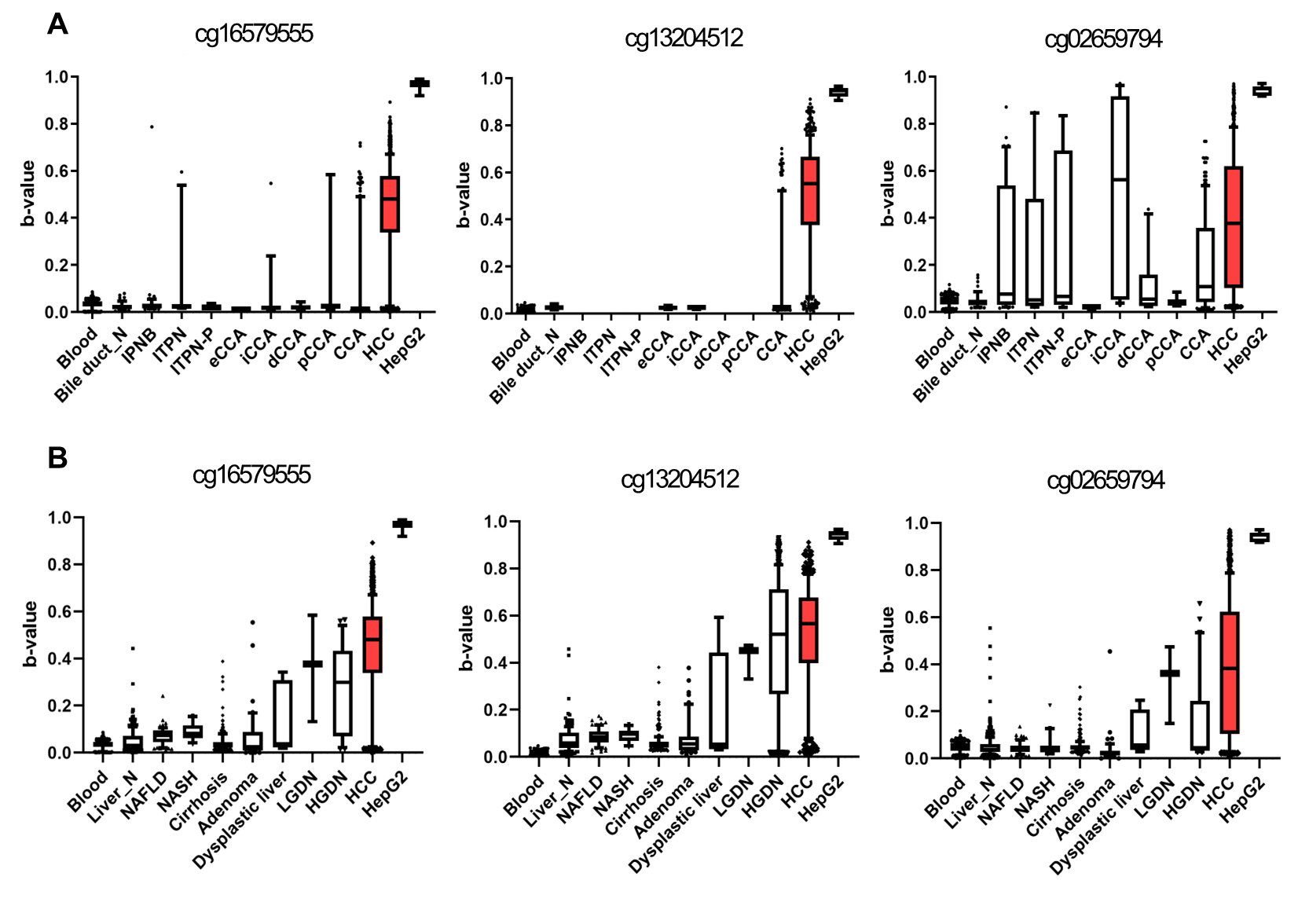


N, normal; IPNB, intraductal papillary neoplasms of the bile duct; ITPN, intraductal tubulopapillary neoplasms of the bile duct; ITPN-P, ITPN of the pancreas; iCCA, intrahepatic cholangiocarcinoma; eCCA, extrahepatic cholangiocarcinoma; dCCA, distal cholangiocarcinoma; pCCA, perihilar cholangiocarcinoma; CCA, cholangiocarcinoma; HCC, hepatocellular carcinoma; NAFLD, non-alcoholic fatty-liver disease; NASH, non-alcoholic steatohepatitis; LGDN, low-grade dysplastic nodules; HGDN, high-grade dysplastic nodules.

**Supplementary Figure 5: Sensitivity Breakdown by HCC Characteristics.**

(A) Clinical sensitivities of the markers in TCGA cohorts are shown according to tumor stage (from T1 to T4), etiology (HBV, HCV, EtOH, NAFLD) and race (White, American, Asian, and Others),

(B) Clinical sensitivities of the markers in the CGRC cohort are shown according to tumor stage (from T1 to T4) and etiology (HBV, HCV, EtOH, NAFLD).


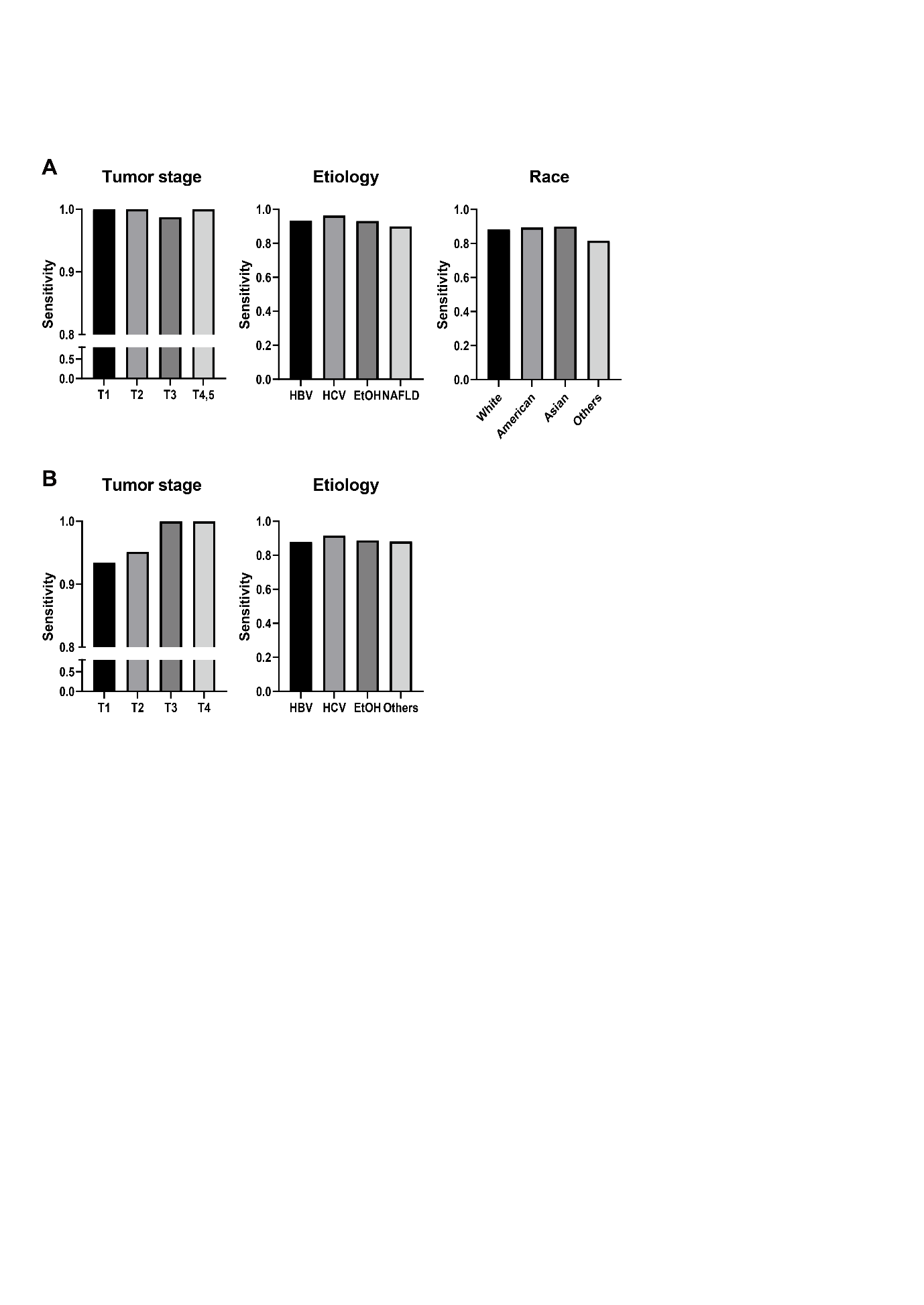


**Supplementary Figure 6: Methylation and marker gene expression correlation.**

Analysis of the correlation between differential methylation value (DM value) of the HCC-specific marker cg16579555 and the differential expression value (Log2 fold change) of its corresponding gene, RNF135 (A) and LDHB (B).

The significance of the correlation is denoted by P, the spearman correlation coefficient is represented by r, and black dot denotes each sample.


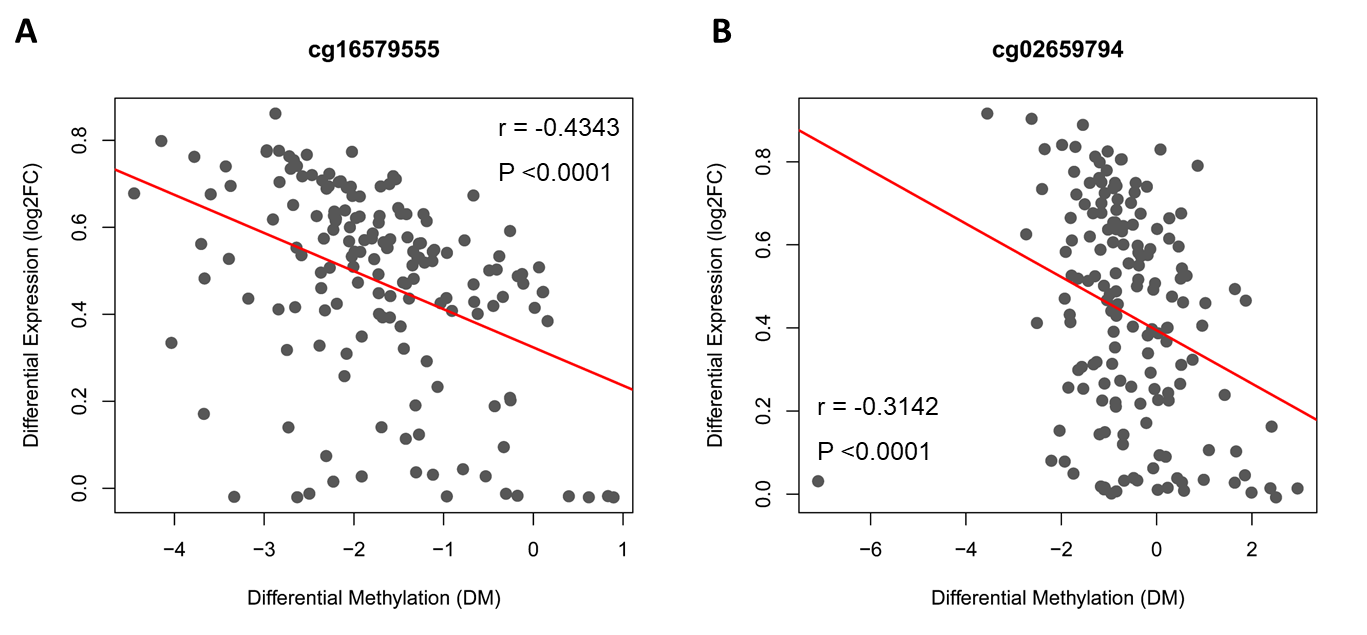


**Supplementary Figure 7: Box plot of the RNF135 and LDHB methylation assay of cfDNAs.**

(A-B) Boxplot representing the distribution of methylation scores for (A) RNF135 and (B) LDHB among the healthy, at-risk, and HCC groups. The x-axis shows the sample, and the y-axis indicates the methylation score, ranging from 0 to 100. A dotted line marks the 95^th^ quantile of the methylation score in the at-risk group (Statistical P values were shown as **, P ≤ 0.01 and ****, P ≤ 0.0001).





**Supplementary Figure 8: Comparing AFP, AFP-L3, GPC3, and MS-HRM assay between HCC and at-risk groups.**

ROC curve demonstrating the diagnostic performance for 304 HCC patients versus 207 at-risk subjects. The color represents the following: AFP (black), AFP-L3 (orange), GPC3 (green), and MS-HRM assay (purple). The x-axis represents 1-specificity, while the y-axis represents sensitivity.


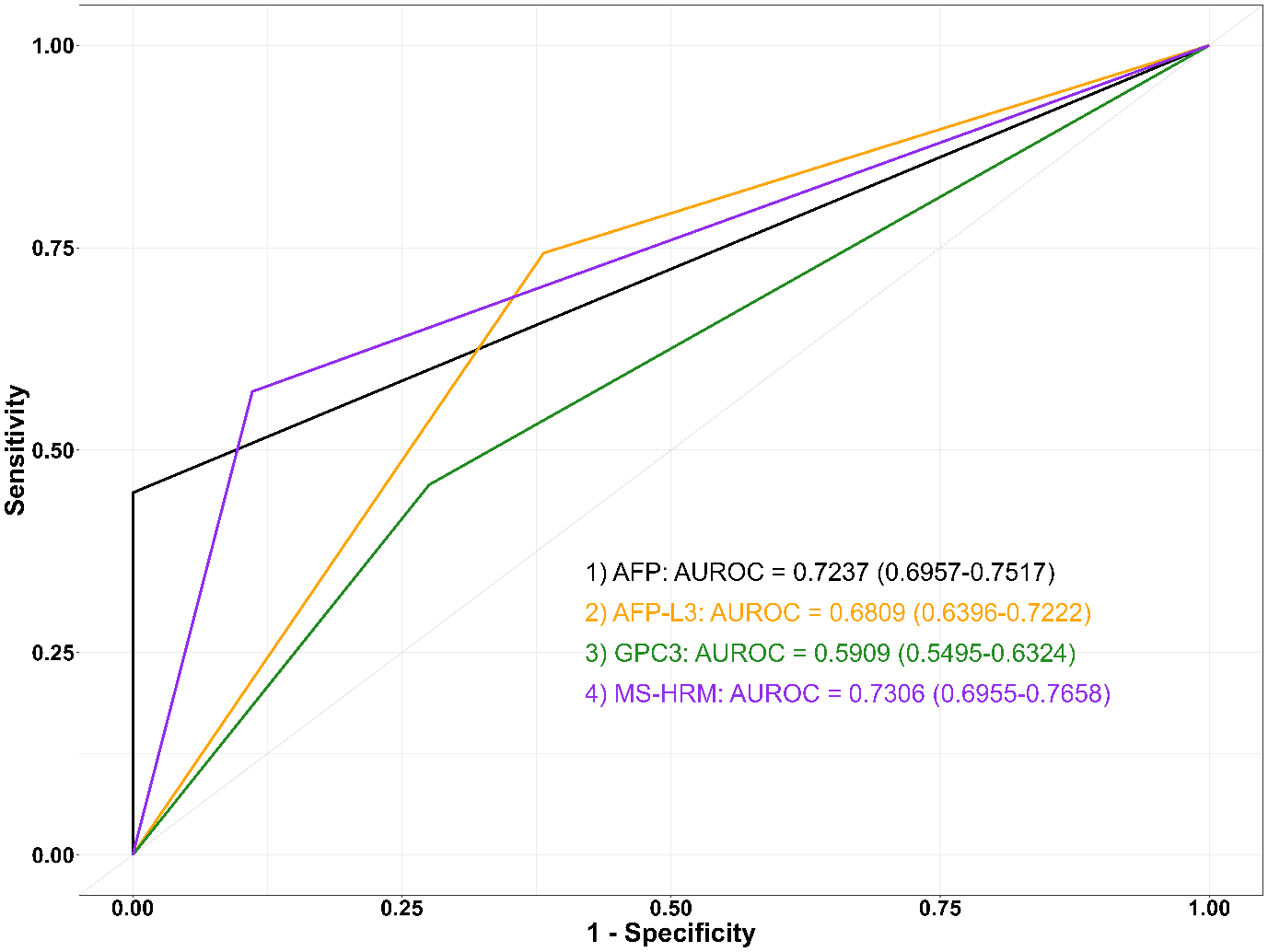


**Supplementary Figure 9: Positive detection rates for both AFP- and GPC3-negative HCC samples.**

The red and gray areas depict the MS-HRM positive and negative rates, respectively, with numbers within each segment denoting the corresponding percentages. The x-axis categorizes stages into early (BCLC 0-A), late (BCLC B-D), and overall stages. The numbers also represent the count of MS-HRM positive samples, and the total samples count in each group.

**
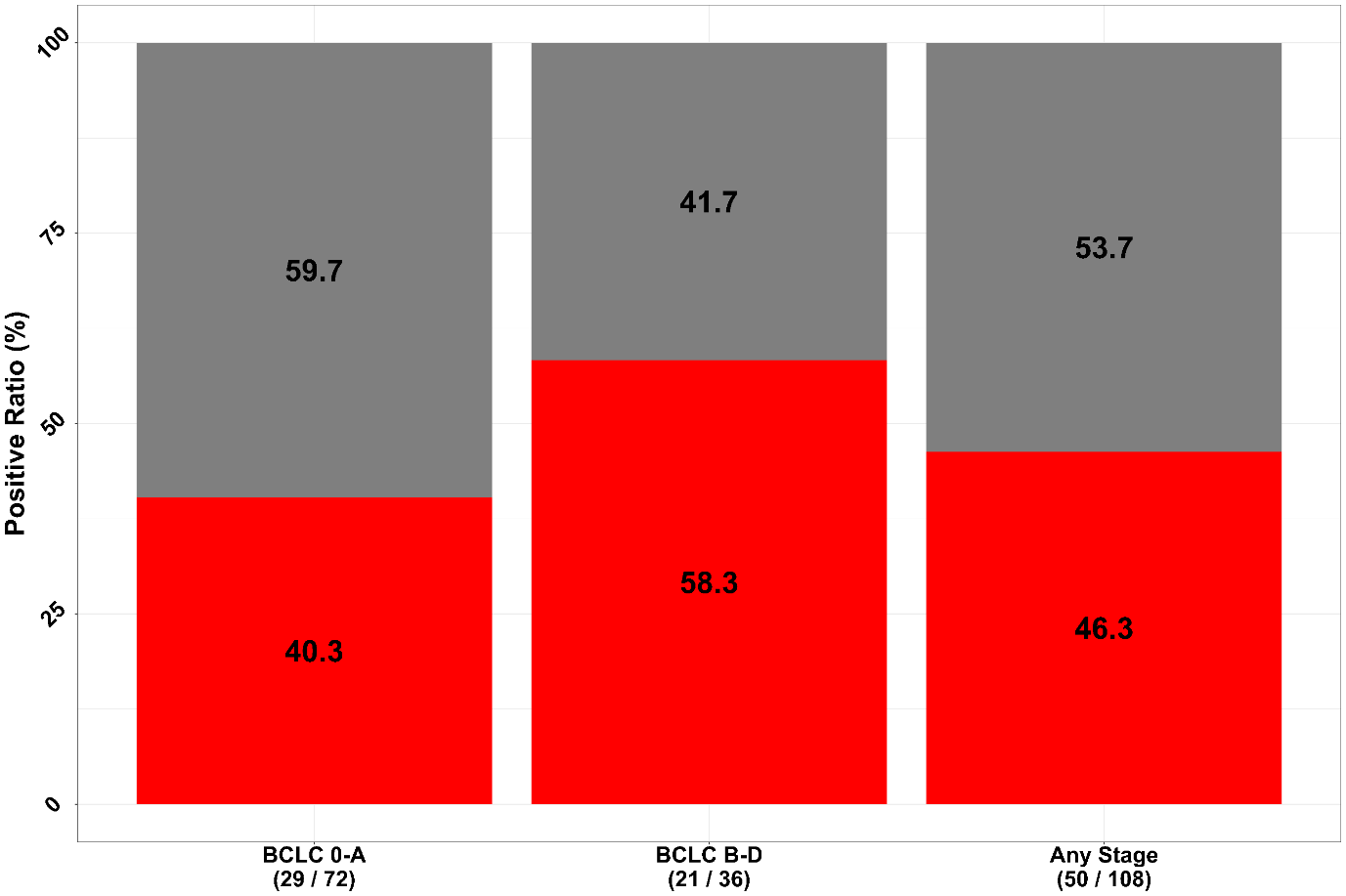
**
